# Endoscopic diagnosis and treatment planning for colorectal polyps using a deep-learning model

**DOI:** 10.1038/s41598-019-56697-0

**Published:** 2020-01-08

**Authors:** Eun Mi Song, Beomhee Park, Chun-Ae Ha, Sung Wook Hwang, Sang Hyoung Park, Dong-Hoon Yang, Byong Duk Ye, Seung-Jae Myung, Suk-Kyun Yang, Namkug Kim, Jeong-Sik Byeon

**Affiliations:** 1 Department of Gastroenterology, University of Ulsan College of Medicine, Asan Medical Center, Seoul, Korea; 2 Department of Convergence Medicine, Asan Medical Institute of Convergence Science and Technology, University of Ulsan College of Medicine, Asan Medical Center, Seoul, Korea; 3 Department of Convergence Medicine and Radiology, Research Institute of Radiology and Institute of Biomedical Engineering, University of Ulsan College of Medicine, Asan Medical Center, Seoul, Korea

**Keywords:** Colonoscopy, Colorectal cancer

## Abstract

We aimed to develop a computer-aided diagnostic system (CAD) for predicting colorectal polyp histology using deep-learning technology and to validate its performance. Near-focus narrow-band imaging (NBI) pictures of colorectal polyps were retrieved from the database of our institution. Of these, 12480 image patches of 624 polyps were used as a training set to develop the CAD. The CAD performance was validated with two test datasets of 545 polyps. Polyps were classified into three histological groups: serrated polyp (SP), benign adenoma (BA)/mucosal or superficial submucosal cancer (MSMC), and deep submucosal cancer (DSMC). The overall kappa value measuring the agreement between the true polyp histology and the expected histology by the CAD was 0.614–0.642, which was higher than that of trainees (n = 6, endoscopists with experience of 100 NBI colonoscopies in <6 months; 0.368–0.401) and almost comparable with that of the experts (n = 3, endoscopists with experience of 2,500 NBI colonoscopies in ≥5 years) (0.649–0.735). The areas under the receiver operating curves for CAD were 0.93–0.95, 0.86–0.89, and 0.89–0.91 for SP, BA/MSMC, and DSMC, respectively. The overall diagnostic accuracy of the CAD was 81.3–82.4%, which was significantly higher than that of the trainees (63.8–71.8%, P < 0.01) and comparable with that of experts (82.4–87.3%). The kappa value and diagnostic accuracies of the trainees improved with CAD assistance: that is, the kappa value increased from 0.368 to 0.655, and the overall diagnostic accuracy increased from 63.8–71.8% to 82.7–84.2%. CAD using a deep-learning model can accurately assess polyp histology and may facilitate the diagnosis of colorectal polyps by endoscopists.

## Introduction

Colonoscopy can effectively detect colorectal polyps of various histological subtypes, including hyperplastic, adenomatous, and malignant polyps. Premalignant polyps, such as adenoma, should be resected endoscopically to prevent their development to colorectal cancer (CRC)^1^. Early CRCs with favorable histological features and cancer invasion up to mucosa or superficial submucosa less than 1,000 μm from the muscularis mucosa can be also managed by endoscopic resection^2^. However, endoscopic resection is not recommended for early CRCs with unfavorable histological features, such as massive submucosal invasion deeper than 1,000 μm, and surgery should be performed for these tumors^3,4^. Diminutive hyperplastic polyps can be left *in situ* without resection because they have no malignant potential, which is called a diagnose-and-leave strategy^5^. Owing to the wide variety of management strategies, the accurate assessment of colorectal polyp histopathology is of crucial importance.

Image-enhanced endoscopy using narrow-band imaging (NBI), blue light imaging, and i-Scan enabled the clear visualization of the microvascular architectures and surface structures of colorectal polyps^6–8^. Systematic classification systems have been developed to predict the histopathology of colorectal polyps based on NBI findings, which include the NBI International Colorectal Endoscopic classification and Japan NBI Expert Team classification^9,10^. Although these classification systems showed good accuracy in the prediction of colorectal polyp histopathology^9^, their performance is greatly endoscopist-dependent and the performance of optical diagnosis in nonacademic centers was disappointing^11^. Therefore, significant learning curves with repetitive training for NBI images are needed to achieve a diagnostic performance with high confidence^12,13^.

Recently, artificial intelligence (AI) has been introduced in an attempt to revolutionize the field of endoscopy. Multi-layered rapid image analysis and feature extraction performed by machine learning, a subset of AI, have been applied in the endoscopic recognition and assessment of colorectal polyps^14–16^. Initial experiences have enabled endoscopists to expect more detailed applications of AI and the innovative shifts in endoscopy practices by overcoming several limitations, including the inter-observer and intra-observer variability of completely endoscopist-dependent practices.

We herein present the development and validation of a computer-aided diagnostic system (CAD) for predicting colorectal polyp histology using AI-based deep learning. We aimed to investigate the possibility of a CAD application in the formation of treatment plans for colorectal polyps.

## Results

### Baseline characteristics of colorectal polyps

The baseline characteristics of 1169 colorectal polyps are presented in Table 1. The median size was 10 mm (range, 2–100 mm). Gross morphology of the Is type was the most common. BA was the most common histological diagnosis (705/1169, 60.3%), while DSMC was the least common (91/1169, 7.8%).

**Table 1:** Baseline characteristics of the included colorectal polyps.

Variables	Overall	Training set	Test set I	Test set II	P*
Number	1169	624	182	363	
Median size, mm (range)	10 (2–100)	8 (2–100)	10 (4–50)	12 (3–90)	0.332
Localization (n, %)					0.407
Right colon	656 (56.1)	334 (53.5)	103 (56.6)	219 (60.3)	
Left colon	513 (43.9)	290 (45.5)	79 (43.4)	144 (39.7)	
Macroscopic type (n, %)					0.825
Ip	42 (3.6)	18 (2.9)	8 (4.4)	16 (4.4)	
Is	820 (70.1)	464 (74.4)	122 (67.0)	234 (64.5)	
LST	307 (26.3)	142 (22.8)	52 (28.6)	113 (31.1)	
LST-NG	140 (12.0)	53 (8.5)	26 (14.3)	61 (16.8)	
LST-G	167 (14.3)	89 (14.3)	26 (14.3)	52 (14.3)	
Pathology (n, %)					0.294
Hyperplastic polyp	93 (8.0)	48 (7.7)	15 (8.2)	30 (8.3)	
Sessile serrated polyp	170 (14.5)	76 (12.2)	24 (13.2)	70 (19.3)	
BA	705 (60.3)	393 (63.0)	106 (58.2)	206 (56.7)	
MSMC	110 (9.4)	62 (9.9)	20 (11.0)	28 (7.7)	
DSMC	91 (7.8)	45 (7.2)	17 (9.3)	29 (8.0)	

LST, laterally spreading tumor; NG, non-granular; G, granular; BA, benign conventional adenoma; MSMC, mucosal or superficial submucosal tumor; DSMC, deep submucosal cancer.

*P values in comparison between the test sets I and II.

### Diagnostic performance of the CAD and comparison with endoscopists

**Figure 1:**
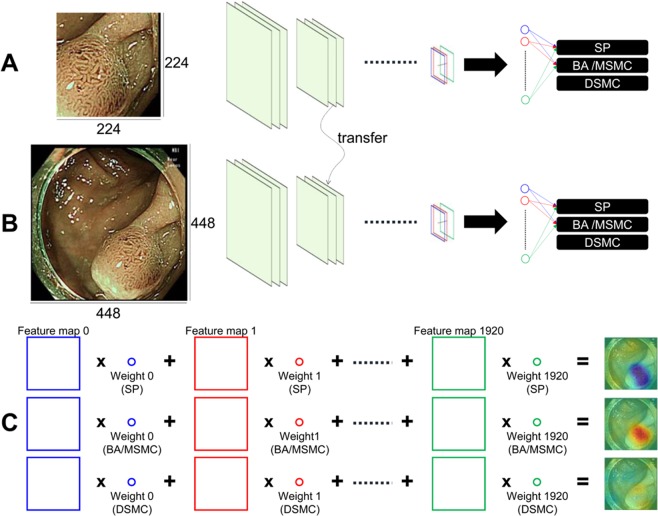
A schematic of the training strategy of the computer-aided diagnostic system (CAD) using a 50-layered convolutional neural network and image patches. SP, serrated polyp; BA, benign conventional adenoma; MSMC, mucosal or superficial submucosal cancer; DSMC, deep submucosal cancer.

The schematic view of the training strategy for the CAD is presented in Fig. 1 and detailed in the Methods section. Among the 182 NBI images of colorectal polyps in test dataset I, the CAD correctly classified 148 images (81.3%). The CAD correctly classified 32 (82.1%) of 39 serrated polyps (SPs), 106 (84.1%) of 126 benign conventional adenoma (BA)/mucosal or superficial submucosal cancer (MSMC) polyps, and 10 (58.8%) of 17 deep submucosal cancer (DSMC) polyps. The overall Cohen’s kappa value for the CAD was 0.614 (95% CI, 0.488–0.730), implying substantial agreement between the actual and predicted histological diagnoses. The Cohen’s kappa value for the trainee endoscopists was 0.368 (95% CI, 0.281–0.459) and that of expert endoscopists was 0.649 (95% CI, 0.564–0.725). Thus, the CAD diagnostic performance was better overall than that of the trainees and comparable to that of expert endoscopists. Detailed kappa values according to polyp size, location, and morphology are presented in Table 2 and show a similar tendency.

**Table 2:** Cohen’s kappa value measuring the agreement between true and predicted histopathological diagnoses in test sets I and II.

Colorectal tumor	Number	Experts		CAD		Trainees		CAD+trainees	
		Kappa	95% CI	Kappa	95% CI	Kappa	95% CI	Kappa	95% CI
**Test set I**									
Overall	182	0.649	0.564–0.725	**0.614**	**0.488–0.730**	0.368	0.281–0.459	0.665	0.560–0.758
Tumor size									
>10mm	78	0.550	0.381–0.685	**0.585**	**0.388–0.749**	0.280	0.117–0.430	0.594	0.405–0.744
≤10mm	104	0.700	0.583–0.800	**0.609**	**0.442–0.757**	0.410	0.300–0.499	0.697	0.547–0.825
Tumor location									
Right colon	103	0.677	0.556–0.775	**0.694**	**0.551–0.822**	0.386	0.286–0.490	0.743	0.614–0.856
Left colon	79	0.602	0.457–0.729	**0.499**	**0.289–0.691**	0.321	0.173–0.455	0.555	0.375–0.716
Tumor morphology									
LST type	52	0.583	0.382–0.752	**0.552**	**0.315–0.769**	0.225	0.038–0.380	0.508	0.262–0.728
Is type	122	0.671	0.564–0.762	**0.645**	**0.490–0.768**	0.436	0.536–0.740	0.740	0.616–0.846
**Test set II**									
Overall	363	0.735	0.690–0.780	**0.642**	**0.552–0.722**	0.401	0.348–0.450	0.658	0.585–0.729
Tumor size									
>10mm	189	0.724	0.650–0.784	**0.623**	**0.494–0.736**	0.416	0.338–0.487	0.674	0.568–0.773
≤10mm	174	0.714	0.637–0.781	**0.620**	**0.495–0.727**	0.300	0.226–0.370	0.599	0.481–0.693
Tumor location									
Right colon	219	0.748	0.687–0.805	**0.596**	**0.485–0.695**	0.355	0.289–0.418	0.623	0.523–0.715
Left colon	144	0.696	0.604–0.776	**0.688**	**0.559–0.800**	0.420	0.326–0.509	0.687	0.575–0.781
Tumor morphology									
LST type	113	0.744	0.654–0.820	**0.633**	**0.474–0.786**	0.385	0.276–0.481	0.684	0.538–0.805
Is type	234	0.712	0.650–0.767	**0.626**	**0.525–0.721**	0.394	0.324–0.455	0.635	0.525–0.710

CAD, computer-aided diagnostic system; CI, confidence interval; LST, laterally spreading tumor.

In test dataset II analyzing the diagnostic performance of the CAD in prospectively acquired real-time NBI images of 363 colorectal polyps, the Cohen’s kappa value for the CAD was also significantly higher than that of trainee endoscopists (0.642 vs. 0.401), while it was comparable or slightly inferior to that of expert endoscopists (0.642 vs. 0.735). Detailed findings are presented in Table 2.

### Diagnostic performance of the CAD in each histological group

The diagnostic performances of the CAD and endoscopists according to the three histological groups are presented in Table 3. In test dataset I, the overall diagnostic accuracy of the CAD was 81.3% compared to that of expert endoscopists being 82.4%, indicating no statistically significant difference. However, the CAD showed significantly better overall diagnostic accuracy compared to the trainee endoscopists (81.3% vs. 71.8%, P = 0.005) (Table 3). Other performance indicators, including sensitivity, specificity, positive predictive value (PPV), and negative predictive value (NPV), were similar between the CAD and the experts in the three histological groups, whereas the performance parameters of the CAD were superior to those of trainees (Table 3).

**Table 3:** Diagnostic performance of the CAD in each histological group in comparison with the diagnostic performance of endoscopists.

All polyps	Test set I							Test set II						
	Experts	CAD	Trainees	CAD+trainees	P*	P^†^	P^‡^	Experts	CAD	Trainees	CAD+trainees	P^*^	P^†^	P^‡^
**Overall accuracy**	82.4 (450/546)	**81.3 (148/182)**	71.8 (392/546)	84.2 (460/546)	0.724	0.005	<0.001	87.3 (951/1089)	**82.4 (299/363)**	63.8 (695/1089)	82.7 (901/1089)	0.005	<0.001	<0.001
**Serrated polyp**	**Experts**	**CAD**	**Trainees**	**CAD+trainees**	P^*^	**P**^**†**^	**P**^**‡**^	**Experts**	**CAD**	**Trainees**	**CAD+trainees**	P^*^	**P**^**†**^	**P**^**‡**^
Sensitivity, % (fraction)	88.9 (104/117)	**82.1 (32/39)**	55.6 (65/117)	82.1 (96/117)	0.179	<0.001	<0.001	81.7 (245/300)	**74.0 (74/100)**	92.0 (276/300)	81.3 (244/300)	0.059	<0.001	0.0003
Specificity, % (fraction)	92.1 (395/429)	**93.7 (134/143)**	90.4 (388/429)	94.9 (407/429)	0.498	0.210	0.057	94.6 (746/789)	**93.5 (246/263)**	61.0 (481/789)	89.2 (704/789)	0.452	<0.001	<0.001
PPV, % (fraction)	75.4 (104/138)	**78.0 (32/41)**	61.3 (65/106)	81.4 (96/118)	0.666	0.018	0.003	85.1 (245/288)	**81.3 (74/91)**	47.3 (276/584)	74.2 (244/329)	0.250	<0.001	<0.001
NPV, % (fraction)	96.8 (395/408)	**95.0 (134/141)**	88.2 (388/440)	95.1 (407/428)	0.193	0.001	0.001	93.1 (746/801)	**90.4 (246/272)**	95.2 (481/505)	92.6 (704/760)	0.046	0.003	0.050
**BA/MSMC**	**Experts**	**CAD**	**Trainees**	**CAD+trainees**	P^*^	**P**^**†**^	**P**^**‡**^	**Experts**	**CAD**	**Trainees**	**CAD+trainees**	P^*^	**P**^**†**^	**P**^**‡**^
Sensitivity, % (fraction)	83.1 (314/378)	**84.1 (106/126)**	81.7 (309/378)	88.1 (333/378)	0.775	0.508	0.040	92.2 (647/702)	**88.5 (207/234)**	53.0 (372/702)	85.8 (602/702)	0.041	<0.001	<0.001
Specificity, % (fraction)	81.0 (136/168)	**75.0 (42/56)**	51.8 (87/168)	75.6 (127/168)	0.304	0.001	<0.001	78.6 (304/387)	**72.1 (93/129)**	84.5 (327/387)	77.8 (301/387)	0.083	<0.001	0.0167
PPV, % (fraction)	90.8 (314/346)	**88.3 (106/120)**	79.2 (309/390)	89.0 (333/374)	0.330	0.002	<0.001	88.6 (647/730)	**85.2 (207/243)**	86.1 (372/432)	87.5 (602/688)	0.043	0.655	0.429
NPV, % (fraction)	68.0 (136/200)	**67.7 (42/62)**	55.8 (87/156)	73.8 (123/172)	0.961	0.028	<0.001	84.7 (304/359)	**77.5 (93/120)**	49.8 (327/657)	75.1 (301/401)	0.016	<0.001	<0.001
**DSMC**	**Experts**	**CAD**	**Trainees**	**CAD+trainees**	P^*^	**P**^**†**^	**P**^**‡**^	**Experts**	**CAD**	**Trainees**	**CAD+trainees**	P^*^	**P**^**†**^	**P**^**‡**^
Sensitivity, % (fraction)	62.7 (32/51)	**58.8 (10/17)**	35.3 (18/51)	58.8 (30/51)	0.839	0.043	0.013	67.8 (59/87)	**62.1 (18/29)**	54.0 (47/87)	63.2 (55/87)	0.532	0.268	0.161
Specificity, % (fraction)	93.9 (465/495)	**93.3 (154/165)**	93.5 (463/495)	92.9 (472/495)	0.771	>0.999	0.678	98.8 (990/1002)	**96.7 (323/334)**	97.4 (976/1002)	98.3 (985/1002)	0.004	0.436	0.201
PPV, % (fraction)	51.6 (32/62)	**47.6 (10/21)**	36.0 (18/50)	46.2 (31/54)	0.655	0.215	0.274	83.1 (59/71)	**62.1 (18/29)**	64.4 (47/74)	76.4 (55/72)	0.004	0.760	0.094
NPV, % (fraction)	96.1 (465/484)	**95.7 (154/161)**	93.3 (463/496)	95.6 (472/492)	0.742	0.114	0.117	97.2 (990/1018)	**96.7 (323/334)**	96.1 (976/1016)	96.9 (985/1017)	0.471	0.324	0.186

CAD, computer-aided diagnostic system; PPV, positive predictive value; NPV, negative predictive value; BA, benign conventional adenoma; MSMC, mucosal or superficial submucosal tumor; DSMC, deep submucosal cancer.

The diagnostic performance of the three experts and trainees was evaluated by combining the results of all the endoscopists. Therefore, the total number of examined polyps was 546 (3 times 182) in test set I and 1089 (3 times 363) in test set II.

^*^P value in the comparison between CAD vs. experts.

^**†**^P value in the comparison between CAD vs. trainees.

^‡^P value in the comparison between trainees vs. CAD+trainees.

In test dataset II, the overall diagnostic accuracy of the CAD was significantly higher than that of trainee endoscopists (82.4% vs. 63.8%, P < 0.001). The CAD showed inferior overall diagnostic accuracy to that of expert endoscopists (82.4% vs. 87.3%, P = 0.005). The analytical results of other performance indicators in test dataset II are presented in Table 3.

**Figure 2:**
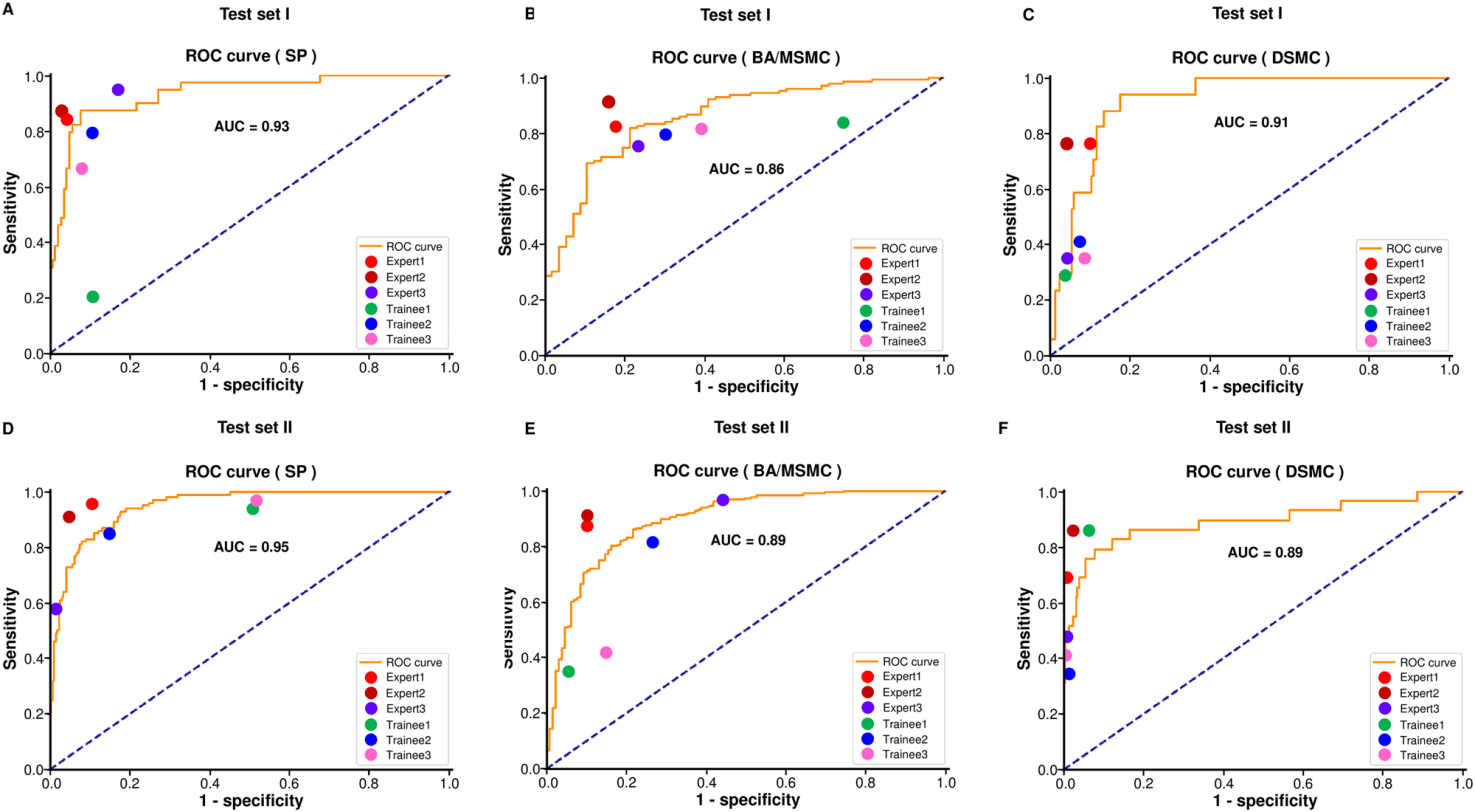
The receiver operating characteristic (ROC) curves evaluating the diagnostic performance of the computer-aided diagnostic system (CAD). The performance of the CAD was evaluated and compared with the performances of three expert endoscopists and three trainees using ROC curves. (**A–C**) The ROC curves for the CAD in the SP, BA/MSMC, and DSMC groups of test dataset I; (**D–F**) The ROC curves for the CAD in the SP, BA/MSMC, and DSMC groups of test dataset II. AUC, area under the ROC curve; SP, serrated polyp; BA, benign conventional adenoma; MSMC, mucosal or superficial submucosal cancer (cancer with invasion depth <1000 µm from the muscularis mucosa); DSMC, deep submucosal cancer (cancer with invasion depth ≥1000 µm from the muscularis mucosa).

Area under the receiver operating characteristic (ROC) curves (AUC) for the CAD showed good-to-excellent diagnostic performances at 0.93, 0.86, and 0.91 for the SP, BA/MSMC, and DSMC groups, respectively, in test dataset I. ROC curves of the CAD in test dataset II showed similar findings (AUCs: 0.95, 0.89, and 0.89 in the SP, BA/MSMC, and DSMC groups, respectively) (Fig. 2). The diagnostic performance of the CAD as demonstrated by the ROC curves was comparable or slightly inferior to that of experts and clearly superior to that of trainees in both test datasets I and II (Fig. 2).

**Figure 3:**
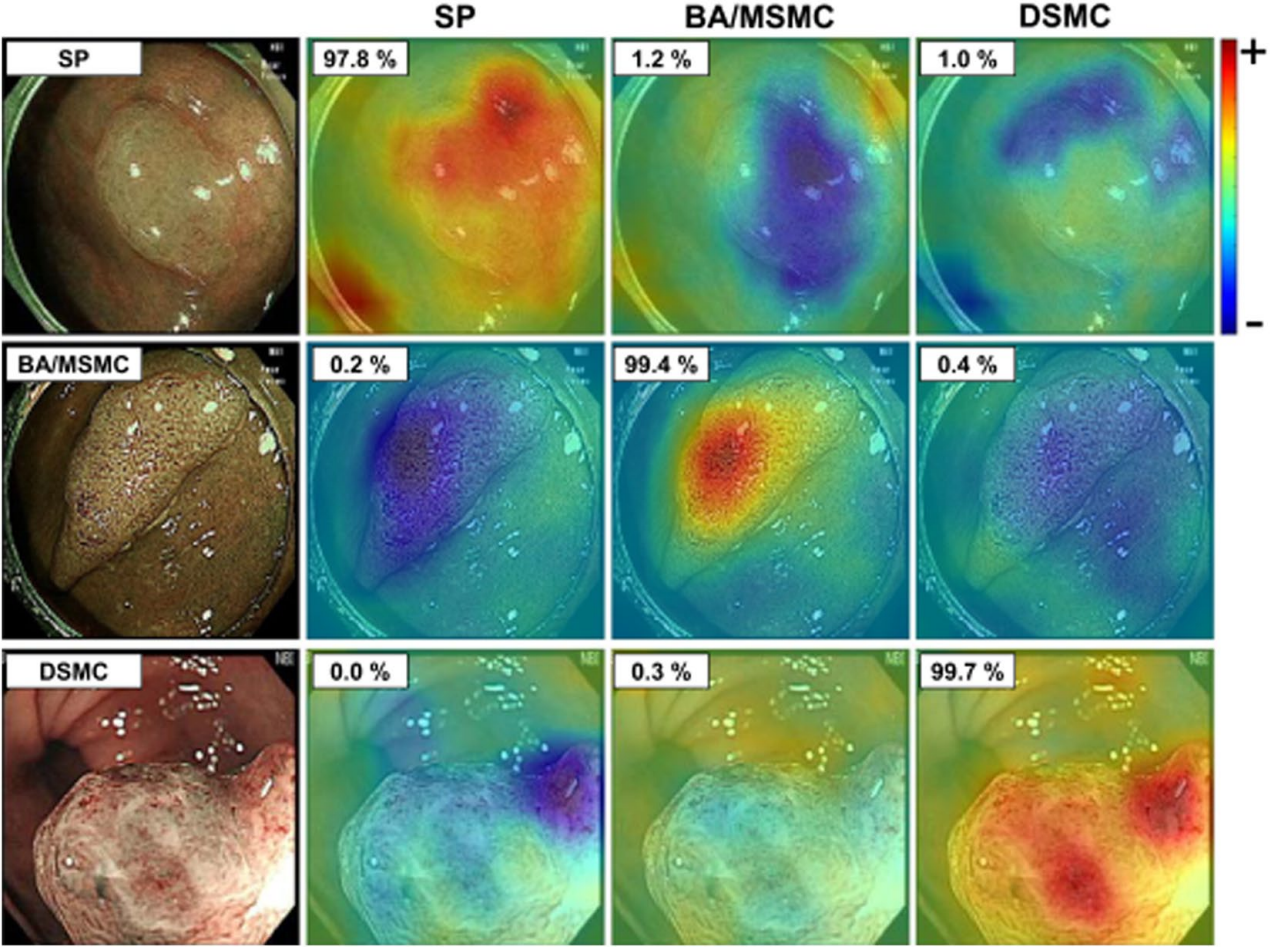
The visualized class activation map images. The figures in small rectangles in each image show the probability of each class being predicted by the computer-aided diagnostic system (CAD). The red area represents the region that the CAD considers to be compatible with the particular histology with high probability. The blue area represents the region that CAD considers to have a low probability for the particular histology. SP, serrated polyp; BA, benign conventional adenoma; MSMC, mucosal or superficial submucosal cancer; DSMC, deep submucosal cancer.

The visualized class activation map images demonstrated that the CAD was able to correctly predict the histological diagnosis of colorectal polyps by perceiving the characteristic surface area of the appropriate, relevant histological group (Fig. 3)^17^.

### Diagnostic performance of trainees assisted by the CAD

**Figure 4:**
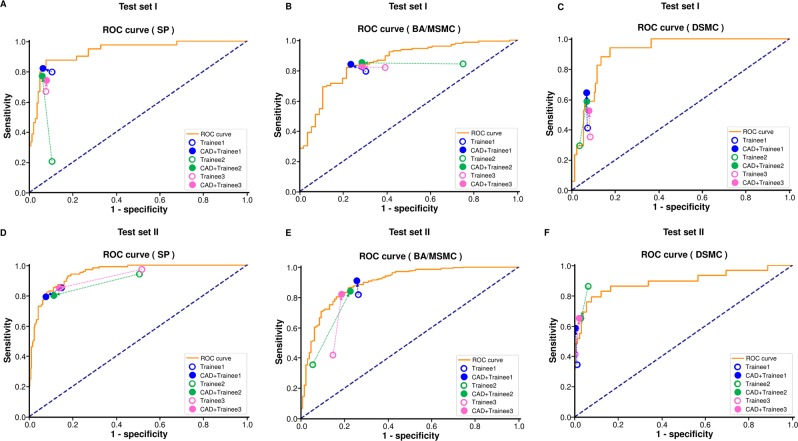
Improvement of the diagnostic performance of trainees with the assistance of the computer-aided diagnostic system (CAD). All empty circles representing trainees’ performance moved to solid circles representing the performance of the CAD+trainees at the left upper side or near the yellow curved line; this suggests that the performance of the CAD+trainees was superior to that of trainees and comparable to that of the CAD (yellow curved line). (**A–C**) Improved diagnostic performance of the CAD+trainee in the SP, BA/MSMC, and DSMC groups of test dataset I; (**D–F**) Improved diagnostic performance of the CAD+trainee in the SP, BA/MSMC, and DSMC groups of test dataset II. SP, serrated polyp; BA, benign conventional adenoma; MSMC, mucosal or superficial submucosal cancer; DSMC, deep submucosal cancer.

In test dataset I, the overall Cohen’s kappa value of the CAD+trainees was 0.665 (95% CI, 0.560–0.758), which was higher than that of the trainees (0.368, 95% CI 0.281–0.459) (Table 2). Moreover, the overall diagnostic accuracy of the CAD+trainees was significantly higher than that of the trainee endoscopists (84.2% vs. 71.8%, respectively; P < 0.001) (Table 3). The ROC curves also showed improvements in the diagnostic performance of the trainees with CAD assistance (Fig. 4).

Analysis of the diagnostic performance of the trainees assisted by the CAD in test dataset II showed similar findings as those in test dataset I (Table 2,3, Fig. 4).

### Diagnostic performance of the CAD according to the size, location, and morphology of colorectal polyps

The diagnostic performance of the CAD in test dataset I was generally good, with no definite, consistent differences according to polyp size (>10 mm vs. ≤10 mm), location (right vs. left colon), or morphology (laterally spreading tumor [LST] vs. Is; Table 4). The diagnostic performance of the CAD according to polyp size, location, and morphology in test dataset II showed tendencies similar to those in test dataset I (Supplementary Table 2).

**Table 4:** Diagnostic performance of the CAD according to the size, location, and morphology of the colorectal polyps in test set I.

Polyp size	Serrated polyp		BA/MSMC		DSMC	
	≤10mm	>10mm	≤10mm	>10mm	≤10mm	>10mm
Accuracy, % (fraction)	85.6 (267/312)	98.7 (231/234)	79.5 (186/234)	79.5 (186/234)	97.1 (303/312)	80.8 (189/234)
Sensitivity, % (fraction)	77.4 (72/93)	100.0 (24/24)	81.5 (132/162)	81.5 (132/162)	0.0 (0/3)	62.5 (30/48)
Specificity, % (fraction)	89.0 (195/219)	98.6 (207/210)	75.0 (54/72)	75.0 (54/72)	98.1 (303/309)	85.5 (159/186)
PPV, % (fraction)	75.0 (72/96)	88.9 (24/27)	88.0 (132/150)	88.0 (132/150)	0.0 (0/6)	52.6 (30/57)
NPV, % (fraction)	90.3 (195/216)	100.0 (207/207)	64.3 (54/84)	64.3 (54/84)	99.0 (303/306)	89.8 (159/177)
**Polyp location**	**Serrated polyp**		**BA/MSMC**		**DSMC**	
	**Right colon**	**Left colon**	**Right colon**	**Left colon**	**Right colon**	**Left colon**
Accuracy, % (fraction)	91.3 (282/309)	91.1 (216/237)	86.4 (267/309)	74.7 (177/237)	95.1 (294/306)	83.6 (198/237)
Sensitivity, % (fraction)	89.3 (75/93)	63.6 (21/33)	86.5 (192/222)	80.8 (126/156)	0.0 (0/3)	62.5 (30/48)
Specificity, % (fraction)	92.0 (207/225)	95.6 (195/204)	86.2 (75/87)	63.0 (51/81)	96.1 (294/306)	88.9 (168/189)
PPV, % (fraction)	80.6 (75/93)	70.0 (21/30)	94.1 (192/204)	80.8 (126/156)	0.0 (0/12)	58.8 (30/51)
NPV, % (fraction)	95.8 (207/215)	94.2 (195/207)	71.4 (75/105)	63.0 (51/81)	99.0 (294/297)	90.3 (168/186)
**Polyp morphology**	**Serrated polyp**		**BA/MSMC**		**DSMC**	
	**LST type**	**Sessile type**	**LST type**	**Sessile type**	**LST type**	**Sessile type**
Accuracy, % (fraction)	96.2 (150/156)	89.3 (327/366)	76.9 (120/156)	82.8 (303/366)	80.8 (126/156)	93.4 (342/366)
Sensitivity, % (fraction)	80.0 (24/30)	85.7 (72/84)	80.0 (84/105)	84.5 (213/252)	57.1 (12/21)	60.0 (18/30)
Specificity, % (fraction)	100.0 (126/126)	90.4 (255/282)	70.6 (36/51)	78.9 (90/114)	84.4 (114/135)	96.4 (324/336)
PPV, % (fraction)	100.0 (24/24)	72.7 (72/99)	84.8 (84/99)	89.9 (213/237)	36.4 (12/33)	60.0 (18/30)
NPV, % (fraction)	95.5 (126/132)	95.5 (225/267)	63.2 (36/57)	69.8 (90/129)	92.7 (114/123)	96.4 (324/336)

CAD, computer-aided diagnostic system; BA, benign conventional adenoma; MSMC, mucosal or superficial submucosal tumor; DSMC, deep submucosal cancer; PPV, positive predictive value; NPV, negative predictive value; LST, laterally spreading tumor.

### Inference time of CAD

The average inference time for histological assessment by the CAD was 0.02 seconds with ResNet-50 and 0.04 seconds with DenseNet-201.

## Discussion

In this study, the CAD based on NBI near-focus images showed a good diagnostic accuracy of >80%–90% irrespective of polyp size, location, and morphology. The area under the ROC curves for the CAD was 0.86–0.95, implying good-to-excellent predictability. The CAD showed a better performance compared to trainees and a slightly inferior or comparable performance to that of experts. In addition, CAD assistance significantly improved the diagnostic performance of trainees. These findings suggest that the AI CAD system helps inexperienced endoscopists to correctly predict the histopathology of colorectal polyps and gives expert endoscopists increased confidence in their histological assessments. Therefore, we believe that CAD assistance will help endoscopists more reliably determine the appropriate treatment plan for colorectal polyps.

Several recent studies have investigated the use of the CAD to differentiate between neoplastic and non-neoplastic lesions, which are summarized in Supplementary Table 3. Our study is distinctive in several aspects. First, the majority of previous studies developed a CAD based on magnifying images or endocytoscopy images; these are not widely available in current clinical practice, particularly in nonacademic hospitals^15,18–22^. In contrast, we developed a CAD using NBI near-focus images that can be easily obtained in many centers, including primary care units. Second, previous CAD studies focused on discriminating only diminutive polyps^14,15,19^. However, appropriate treatment plans for both large and small colorectal polyps are essential to achieve successful screening and surveillance colonoscopy. Therefore, we enrolled all colorectal polyps regardless of their size and trained the CAD to classify the polyps into three histological groups with varying treatment plans. The BA/MSMC group is endoscopically resectable, while the DSMC group is endoscopically unresectable group and requires surgery. Since differentiating between hyperplastic and sessile serrated polyps is difficult even through histological evaluation by pathologists^23,24^, the treatment plan for SP is currently determined clinically based on the size and location of the SP; endoscopic resection is recommended for SP > 5 mm at any location and SP of any size at a location proximal to the sigmoid colon. Accordingly, real-time differentiation between the two types is not mandatory in current practice. Therefore, we suggest that our three histological groups adequately represented the treatment planning of most colorectal polyps, and our study showed that the CAD is a potentially good modality to aid in the differentiation of these three histological groups.

Interestingly, the diagnostic performance of trainees improved significantly with CAD assistance through our heuristic algorithm. We suggest that this type of assessment considering both the endoscopist’s confidence level and the CAD probability may be a good way to implement AI CAD into real clinical practices. If an AI device shows a clearly superior performance, it could entirely replace human clinicians. However, consensus on the definition of “clear superiority” is difficult to achieve. Therefore, approaches combining both human and AI-suggested decisions can be a practical solution, and we believe our algorithm shows such an example of colonoscopy practices using a CAD.

The class activation map images showed the correct perception of the representative surface area of colorectal polyps by the CAD (Fig. 3). In general, the deep-learning model is regarded as a black box function because it is a data-driven method without inference by well-defined scientific laws. However, if the inference by the model cannot be interpreted, it would not be useful in clinical practice. As part of the solution to this problem, the class activation map can be extracted for the inference reason of the model. As shown in Fig. 3, the class activation map indicated that the model inferred histopathological diagnoses by correct perception of the characteristic polyp surface similarly to the endoscopists.

One disappointing aspect of our study was the relatively unsatisfactory performance of the CAD in the DSMC group. The CAD correctly classified only 10 (58.8%) of 17 DSMC polyps in test set I and 18 (62.1%) of 29 DSMC polyps in test set II. This may be partly related to the small number of DSMC cases. Another possibility is that only a single NBI near-focus image in test set I and 1–5 NBI near-focus images in test set II may not represent all the features of the entire tumor, particularly for DSMC. A larger DSMC dataset and diagnosis based on multiple images for each DSMC may be warranted in future studies.

This study has several limitations. First, our CAD predicted colorectal polyp histology based on still images, and investigation of an AI CAD system assessing motion images should be developed and validated. Nonetheless, because the images used for the CAD training in this study were unmodified from the original images, we are optimistic about the application of motion images to the CAD. In addition, we assessed the performance of the CAD in two separate test datasets. Of those, in test dataset II, real-time assessment of histological diagnoses was performed after the instant transmission of still images to the CAD. We believe this experiment showed the feasibility of real-time assessment even with still images in clinical practice. Second, all NBI near-focus pictures in this study were taken by expert endoscopists. Inexperienced endoscopists may not pinpoint the representative region of a given colorectal polyp, thereby decreasing the performance of the CAD. Despite these limitations, our study is significant in that we demonstrated the possibility of using an AI CAD as a real-time histological diagnostic tool for not only small but also large colorectal polyps, including early cancers. A short inference time of only 0.02–0.04 seconds by our CAD is another important factor in the clinical utility of this system, because rapid diagnosis is mandatory in daily practice.

In conclusion, a CAD developed using a deep-learning model accurately predicted colorectal polyp histology based on NBI images with high accuracy. The diagnostic performance of the CAD was comparable to that of expert endoscopists and better than that of trainees. Real-time histological assessment of colorectal polyps by the CAD may enhance endoscopists’ decision-making and confidence in the selection of appropriate treatment plans.

## Methods

### Patients and data collection

We collected NBI near-focus images of endoscopically resected colorectal polyps at Asan Medical Center between 2014 and 2018. All endoscopic images, including both white light and NBI, were taken using CF-H290 colonoscopes (Olympus Co, Tokyo, Japan). The exclusion criteria were as follows: (1) colorectal polyps without NBI near-focus images, (2) those with dirty mucus and/or feces on their surface, (3) out-of-focus images, and (4) images with evident motion blurring. First, we retrospectively collected 806 NBI near-focus images of 806 polyps, with one image per polyp, in 646 patients. Among these, 624 images were used as the training dataset and 182 were used as the test dataset I. Second, we prospectively collected NBI near-focus images of colorectal polyps as the test dataset II for another separate real-time performance test of the developed CAD system. The same exclusion criteria as those described above for the training dataset and test dataset I were applied. The test dataset II included 546 near-focus images of 363 colorectal polyps, with 1–5 images per each polyp, in 305 patients. Finally, a total of 1352 NBI near-focus images of 1169 colorectal polyps in 951 patients were collected.

This study was approved by the institutional review board (IRB) of Asan Medical Center (2017–1357). Due to the retrospective study design, written informed consent was not obtained from participants. The IRB of our institution waived the need for informed consent based on the non-invasive and anonymized nature of this study. This study was conducted in accordance with institutional ethical guidelines and the Declaration of Helsinki.

### Histopathological classification of colorectal polyps

The histopathology of all colorectal polyps was evaluated by board-certified gastrointestinal pathologists. The polyps were classified into three histological groups: (1) SP, (2) BA/ MSMC, and (3) DSMC. The SP group encompassed hyperplastic and sessile serrated polyps. Superficial submucosal cancer was defined as cancer with an invasion depth <1000 µm from the muscularis mucosa. DSMC was defined as cancer with an invasion depth ≥1000 µm from the muscularis mucosa.

### Development of the CAD

A deep-learning model was used to develop a CAD. ResNet-50^25^ and DenseNet-201^26^ models with proven performance in the ImageNet Large-Scale Visual Recognition Competition (ILSVRC)^27^ were used as a deep-learning architecture to train the weak supervisions of histological diagnoses of NBI near-focus images. ResNet-50 was initially adopted, and then the recently introduced DenseNet-201 was used to improve the performance of the CAD.

Among the retrospectively collected NBI near-focus images of 806 polyps, 624 were used as the training dataset and 182 were used as the test dataset I. Since the prediction of a single NBI near-focus image of a tumor could be easily overfitted in small data-intensive situations, we employed a simple curriculum learning strategy. The tumor area of the collected NBI near-focus images was denoted with a rectangle. Then, 20 half-size image patches containing the center point of these evidences were extracted from each entire image. Through this process, 12480 image patches measuring 224 × 224 were extracted from 624 entire images of 448 × 448 size in the training set. In order to pre-train image patches and fine-tune the model using entire images, two steps of training were performed as shown in Fig. 1. First, the model that was pre-trained on the ILSVRC dataset was trained using an augmented dataset of 12480 image patches as the training data. Thereafter, it was fine-tuned using 624 entire images. This curriculum learning strategy was intended to lead the model to a better local minimum.

There was an imbalance in the number of datasets among the SP, BA/MSMC, and DSMC groups. Since this imbalance could have led to paradoxical outcome, an oversampling strategy was employed to extract the same number of samples per training epoch. Each sample was standardized and trained using common data augmentation techniques, such as adding Gaussian noise, rotating, zooming, and shifting.

All experiments were implemented in Keras with a Tensorflow backbone; a stochastic gradient descent optimizer^28^ was used with 5e^−5^ learning and 5e^−5^ decay rates.

Since the model was validated using the test set without a separate validation set, the reliability of the model’s performance was assessed by 5-fold cross-validation. Supplementary Table 1 shows the cross-validation results for ResNet-50 and DenseNet-201. The mean values of the accuracies were 77.4 for ResNet-50 and 81.4 for DenseNet-201 (P = 0.08). Although there was no statistically significant difference, DenseNet-201 showed a numerically higher accuracy. Therefore, the final CAD system was developed using DenseNet-201.

### Diagnostic performance of the CAD and comparison with endoscopists

The diagnostic performance of the CAD was tested twice separately. The first test was conducted with NBI near-focus images of 182 colorectal polyps in test dataset I. Diagnosis by the CAD was made based on the probabilities of the three histological groups. For example, if the CAD showed a 5% probability for SP, 10% probability for BA/MSMC, and 85% probability for DSMC, the diagnosis was finalized as DSMC, as it had the highest probability. Diagnostic accuracy, sensitivity, specificity, PPV, and NPV were investigated in the differential diagnosis among SP, BA/MSMC, and DSMC. Diagnostic performances were further assessed according to polyp size, morphology, and location. Polyp morphology was classified into Ip (pedunculated), Is (sessile), and LST. LSTs were further categorized into granular (LST-G) and non-granular (LST-NG) types. Tumors were also categorized based on location as follows: tumors in the left or right colon (above the splenic flexure).

To compare the CAD performance with that of endoscopists, six endoscopists blinded to the histological diagnoses were asked to classify the same 182 polyps of the test dataset I into three histological groups based on NBI near-focus images. Endoscopists provided their diagnosis with a confidence level (high vs. low). Three of the six endoscopists were board-certified expert colonoscopists who had experienced approximately 2,500 NBI colonoscopies in ≥5 years. The other three were trainees who had experienced approximately 100 NBI colonoscopies in < 6 months.

We also evaluated the diagnostic performance of trainees assisted by the CAD (CAD+trainee). The final diagnosis by the CAD+trainee was made according to the following algorithm: (1) If the CAD and trainee made the same diagnosis, it was considered the final diagnosis of the CAD+trainee; (2) if the CAD and trainee diagnoses were different and the diagnostic probability by the CAD was ≥80%, the CAD diagnosis was considered the diagnosis of CAD+trainee; (3) if the CAD and trainee diagnoses were different, the diagnostic probability by the CAD was <80%, and the confidence level of the trainee diagnosis was high, the trainee’s diagnosis was considered the diagnosis of the CAD+trainee; (4) if the CAD and trainee diagnoses were different, the diagnostic probability by the CAD was <80%, and the confidence level of trainee diagnosis was low, the CAD diagnosis was considered the diagnosis of the CAD+trainee. A diagnostic probability of 80% is a heuristic parameter that can be changed depending on the model.

The second test for the diagnostic performance of the CAD was performed with NBI near-focus images of 363 colorectal polyps in test dataset II. To test the real-time performance, the following steps were performed. First, 1–5 representative NBI near-focus still images of a colorectal polyp were acquired during colonoscopy. Second, the image was transmitted to the CAD on a laptop via the picture archiving and communication system (PACS) of our center. Finally, the CAD determined the histological diagnostic group in real-time. The same three expert endoscopists and another three trainees assessed the histological diagnoses of the 363 colorectal polyps in test dataset II after all the NBI near-focus images were collected.

### Statistical analysis

Continuous variables were expressed as medians with ranges and categorical variables as frequencies with percentages. We used the unpaired Student’s *t*-test or Mann–Whitney U-test to compare continuous variables and the chi-square or Fisher’s exact test to compare categorical variables. The agreement between the true and predicted histological diagnoses was evaluated using Cohen’s kappa coefficient. The average of the kappa values for each endoscopist was calculated, and 95% CI was estimated by the percentile bootstrap method based on 1000 resamples. Sensitivity, specificity, accuracy, PPV, and NPV were compared using logistic regression with generalized estimating equations that accounted for the clustering of the same patient. A ROC curve was generated to evaluate the diagnostic performance in each histological group. *P* < 0.05 was considered statistically significant. All statistical analyses were performed using SPSS ver. 21.0 for Windows (IBM SPSS; IBM Co., NY, USA) and SAS (version 9.4; AS Institute, Cary, NC, USA).

## Supplementary information

Supplementary Dataset 1.
