# Supplementary material for: Endoscopic diagnosis and treatment planning for colorectal polyps using a deep-learning model
Source: Sci Rep. 2020 Jan 8;10:30. doi: 10.1038/s41598-019-56697-0 (PMC6949236; doi:10.1038/s41598-019-56697-0)
Supplement: Supplementary file 1 — Supplementary Dataset 1. [file 41598_2019_56697_MOESM1_ESM.docx]

**Endoscopic diagnosis and treatment planning for colorectal polyps using a deep-learning model**

**Eun Mi Song**^1^**^†^**, **Beomhee Park**^2^**^†^**, Chun-Ae Ha^1^, Sung Wook Hwang^1^, Sang Hyoung Park^1^, Dong-Hoon Yang^1^, Byong Duk Ye^1^, Seung-Jae Myung^1^, Suk-Kyun Yang^1^, **Namkug Kim**^3^* and **Jeong-Sik Byeon**^1^*

^1^Department of Gastroenterology, University of Ulsan College of Medicine, Asan Medical Center, Seoul, Korea

^2^Department of Convergence Medicine, Asan Medical Institute of Convergence Science and Technology, University of Ulsan College of Medicine, Asan Medical Center, Seoul, Korea

^3^Department of Convergence Medicine and Radiology, Research Institute of Radiology and Institute of Biomedical Engineering, University of Ulsan College of Medicine, Asan Medical Center, Seoul, Korea

**^†^**These two authors contributed equally as the first authors.

^*^These two authors contributed equally as the corresponding authors.

**Supplementary Table 1. Five-fold cross-validation of two models**

|  | Five-fold cross-validation (%) | | | |  |
| --- | --- | --- | --- | --- | --- |
|  | Mean | SD | Min | Max | P-value |
| ResNet-50 | 77.4 | 1.4 | 75.8 | 79.7 | 0.08 |
| DenseNet-201 | 81.4 | 3.7 | 75.7 | 87.3 |  |

**Supplementary Table 2. Diagnostic performance of the CAD according to the size, location, and morphology of the colorectal polyps in test set II**

| Polyp size | Serrated polyp | | BA/MSMC | | DSMC | |
| --- | --- | --- | --- | --- | --- | --- |
|  | ≤10 mm | >10 mm | ≤10 mm | >10 mm | ≤10 mm* | >10 mm |
| Accuracy,  % (fraction) | 81.6 (142/174) | 94.1 (178/189) | 82.2 (143/174) | 83.1 (157/189) | _ | 88.9 (168/189) |
| Sensitivity,  % (fraction) | 75.0 (54/72) | 71.4 (20/28) | 86.3 (88/102) | 90.2 (119/132) | _ | 62.1 (18/29) |
| Specificity,  % (fraction) | 86.3 (88/102) | 98.1 (158/161) | 76.4 (55/72) | 66.7 (38/57) | _ | 93.8 (150/160) |
| PPV,  % (fraction) | 79.4 (54/68) | 87.0 (20/23) | 83.8 (88/105) | 86.2 (119/138) | _ | 64.3 (18/28) |
| NPV,  % (fraction) | 83.0 (88/106) | 95.2 (158/166) | 79.7 (55/69) | 74.5 (38/51) | _ | 93.2 (150/161) |
| Polyp location | Serrated polyp | | BA/MSMC | | DSMC | |
|  | Right colon | Left colon | Right colon | Left colon | Right colon | Left colon |
| Accuracy,  % (fraction) | 83.6 (183/219) | 95.1 (137/144) | 80.8 (177/219) | 85.4 (123/144) | 96.3 (211/219) | 90.3 (130/144) |
| Sensitivity,  % (fraction) | 72.2 (57/79) | 80.9 (17/21) | 87.3 (117/134) | 90.0 (90/100) | 33.3 (2/6) | 69.6 (16/23) |
| Specificity,  % (fraction) | 90.0 (126/140) | 97.6 (120/123) | 70.6 (60/85) | 75.0 (33/44) | 98.1 (209/213) | 94.2 (114/121) |
| PPV,  % (fraction) | 80.3 (57/71) | 85.0 (17/20) | 82.4 (117/142) | 89.1 (90/101) | 33.3 (2/6) | 69.6 (16/23) |
| NPV,  % (fraction) | 85.1 (126/148) | 96.8 (120/124) | 77.9 (60/77) | 76.7 (33/43) | 98.1 (209.213) | 94.2 (114/121) |
| Polyp morphology | Serrated polyp | | BA/MSMC | | DSMC | |
|  | LST type | Sessile type | LST type | Sessile type | LST type | Sessile type |
| Accuracy,  % (fraction) | 92.9 (105/113) | 85.0 (199/234) | 85.0 (96/113) | 80.8 (189/234) | 92.0 (104/113) | 94.5 (222/234) |
| Sensitivity,  % (fraction) | 62.5 (10/16) | 76.2 (64/84) | 90.5 (76/84) | 86.6 (116/134) | 76.9 (10/13) | 50.0 (8/16) |
| Specificity,  % (fraction) | 97.9 (95/97) | 90.0 (135/150) | 69.0 (20/29) | 73.0 (73/100) | 94.0 (94/100) | 98.2 (214/218) |
| PPV,  % (fraction) | 83.3 (10/12) | 81.0 (64/79) | 89.4 (76/85) | 81.1 (116/143) | 62.5 (10/16) | 66.7 (8/12) |
| NPV,  % (fraction) | 94.1 (95/101) | 87.1 (135/155) | 71.4 (20/28) | 80.2 (73/91) | 96.9 (94/97) | 96.4 (214/222) |

CAD, computer-aided diagnostic system; BA, benign conventional adenoma; MSMC, mucosal or superficial submucosal tumor; DSMC, deep submucosal cancer; PPV, positive predictive value; NPV, negative predictive value; LST, laterally spreading tumor

*There was no DSMC case that was ≤10 mm in diameter.

**Supplementary Table 3. Summary of studies evaluating the usefulness of CAD in differential diagnosis of colorectal polyps**

|  | Image | Comparison | Outcome measures | Data sets | Outcomes |
| --- | --- | --- | --- | --- | --- |
| Gross et al. 2011^20^ | Magnified NBI images | CAD vs. experts vs. non-experts | Accuracy in identifying non-neoplastic and neoplastic lesion in small polyps (< 10 mm) | 434 small colorectal polyps (258 neoplastic and 176 non-neoplastic polyps) from 214 patients | Overall diagnostic accuracy: 93.1%  Superior to non-experts and comparable to experts. |
| Takemura et al. 2012^19^ | Magnified NBI images  (ROI for analysis) | CAD vs. experts | Accuracy in identifying Hiroshima type A and B-C3 | 371 colorectal lesions (324 neoplastic and 47 non-neoplastic lesions) | Overall diagnostic accuracy: 97.8%  Diagnostic concordance with experts: 98.7% |
| Mori et al. 2015^21^ | Endocytoscopic images | CAD vs. experts vs. non-experts | Accuracy in identifying neoplastic and non-neoplastic lesion in small polyps (≤ 10 mm) | 176 small colorectal polyps (127 neoplastic and 39 non-neoplastic polyps) from 152 patients | Overall diagnostic accuracy: 89.2%  Superior to non-experts, but inferior to experts. |
| Mori et al. 2016^18^ | Endocytoscopic images | CAD vs. experts vs. non-experts | Accuracy in identifying neoplastic and non-neoplastic lesion in small polyps (≤ 10 mm) | 205 small colorectal polyps (147 neoplastic and 58 non-neoplastic polyps) from 123 patients | Overall diagnostic accuracy: 89% (95% CI, 83-94%)  Superior to non-experts and comparable to experts. |
| Kominami et al. 2016^22^ | Real-time magnified NBI images  (ROI for analysis) | Performance of CAD | Accuracy in identifying Hiroshima type A and B-C3 | 118 colorectal lesions (73 neoplastic and 45 non-neoplastic lesions) from 41 patients | Overall diagnostic accuracy: 94.9% |
| Byrne et al. 2017^14^ | Real time NBI images | Performance of CAD | Accuracy in identifying hyperplastic polyp and neoplastic lesion in diminutive polyps (< 5 mm) | 125 videos of diminutive colorectal polyps (74 adenomas and 51 hyperplastic polyps) | Overall diagnostic accuracy: 94% (95% CI, 86-97%) |
| Chen et al. 2018^15^ | Magnified NBI images | CAD vs. experts vs. non-experts | Accuracy in identifying hyperplastic polyp and neoplastic lesion in diminutive polyps (< 5 mm) | 284 diminutive colorectal polyps (188 neoplastic and 96 hyperplastic polyps) from 193 patients | Overall diagnostic accuracy: 90.1%  Superior to non-experts and comparable to experts. |

CAD, computer-aided diagnostic system; ROI, region of interest; CI, confidence interval
